# Immunostaining of intact *C. elegans* using polyacrylamide embedding

**DOI:** 10.1016/j.xpro.2022.101956

**Published:** 2023-02-09

**Authors:** Abbas Ghaddar, Wenfan Ke, Eyleen J. O’Rourke

**Affiliations:** 1 University of Virginia, Department of Biology, Charlottesville, VA 22903, USA; 2 University of Virginia School of Medicine, Department of Cell Biology, Charlottesville, VA 22903, USA; 3 Robert M. Berne Cardiovascular Research Center, School of Medicine, University of Virginia, Charlottesville, VA 22903, USA

**Keywords:** Cell Biology, Developmental biology, Microscopy, Model Organisms, Molecular Biology, Antibody

## Abstract

A major barrier to immunostaining *Caenorhabditis elegans* is the permeabilization of the worm's cuticle without distorting or damaging its body. We present here a gel-based immobilization protocol for fixed worms coupled with chemical and enzymatic permeabilization. The permeabilization is followed by antibody staining and fluorescent imaging. This protocol can be modified for different fixatives, permeabilizing reagents, or molecular readouts. Unlike previous immunostaining approaches, such as freeze cracking or dissection, this protocol enables immunostaining across the whole body of a well-preserved *C. elegans*.

## Highlights

• Optimized protocol for antibody staining of whole *C. elegans*
• Gel-based immobilization of *C. elegans* on microscope slides
• Permeabilization approach that maintains the integrity of the *C. elegans* body
• Protocol is compatible with other readouts (dyes, RNA probes)

## Abstract (editor highlights)

Publisher’s note: Undertaking any experimental protocol requires adherence to local institutional guidelines for laboratory safety and ethics.

## Graphical abstract

**Figure:**
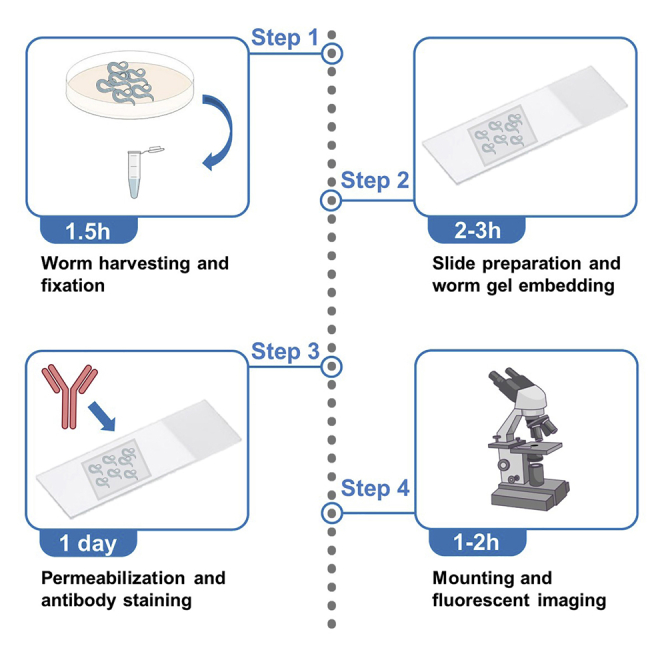

## Before you begin

For guidance into the growth and maintenance of *Caenorhabditis elegans*, please refer to the WormBook article entitled “Maintenance of *C. elegans*”.^1^

The protocol is demonstrated here by staining worms with a custom made anti-LGG-1 antibody (Company: Covalab). However, we have successfully used this protocol with a commercial antibody (anti-GFP, Invitrogen, Cat. #A11122). Immunostaining against other proteins may require adjusting the concentration and time of incubation of primary and secondary antibodies. Additionally, having overcome the critical barriers (immobilization and permeabilization), this protocol is expected to enable labeling different molecules, including the use of dyes and other probes.

### Prepare the required buffers and materials

**Timing: 1 h**

S-buffer, PBS, Tris-HCl (pH 7.5), 2% NaN3, 0.5 M EDTA, 0.5% PBS-T, 60% isopropanol, and Bind-Silane pre-solution can all be prepared ahead of time. Please refer to the “materials and equipment” section for recipes.

## Key resources table

**Table:** 

REAGENT or RESOURCE	SOURCE	IDENTIFIER
**Chemicals, peptides, and recombinant proteins**		

Sodium chloride	Fisher Scientific	Cat. #S671-500
Monopotassium phosphate	Fisher Scientific	Cat. #P285-3
Disodium phosphate	Fisher Scientific	Cat. #S375-500
Potassium hydroxide	VWR	Cat. #BDH7212-1
40% Acrylamide/Bis solution 19:1	Bio-Rad	Cat. #1610144
Tris base	Fisher Scientific	Cat. #T395-500
Hydrochloric acid	Fisher Scientific	Cat. #SA48-1
Tween-20	Sigma	Cat. #P9416-100ML
200 proof ethanol	KOPTEC	Cat. #V1016
Glacial acetic acid	Fisher Scientific	Cat. #A38-500
Bind-silane	GE Healthcare	Cat. #17-1330-01
Repel-silane	GE Healthcare	Cat. #17-1332-01
Sigmacote	MilliporeSigma	Cat. #SL2-25ML
Ammonium persulfate (APS)	Thermo Scientific	Cat. #17874
Tetramethylethylenediamine (TEMED)	Fisher BioReagents	Cat. #BP150-20
β-Mercaptoethanol	MP Biomedicals	Cat. #806443
Triton X-100	Sigma	Cat. #X100-5ML
Calcium chloride	Fisher Scientific	Cat. #S25221A
Collagenase type I	Sigma	Cat. #C0130-100MG
Bovine serum albumin (BSA)	Lee BioSolutions	Cat. #100-10
Sodium azide	Sigma	Cat. #S-2002-25G
EDTA	Fisher	Cat. #BP118-500

**Experimental models: Organisms/strains**		

*C. elegans*	*C. elegans* Genetics Center	N2

**Other**		

PTFE slides	Fisher Scientific	Cat. #10028210
Standard microscope slides	Thermo Scientific	Cat. #3011
Coverslips	Fisher Scientific	Cat. #12-541-B
Vacuum desiccator	SP Scienceware	Zoro #: G4673182
CultureWell silicon gaskets	Grace Bio-Labs	SKU: 103230
Microcentrifuge	N/A	N/A

## Materials and equipment

**Table:** 10× Phosphate-buffered saline

Reagent	Final concentration	Amount
**First mix the following reagents**		

Sodium chloride	1.37 M	80 g
Potassium chloride	27 mM	2 g
Disodium phosphate	100 mM	14.4 g
Monopotassium phosphate	18 mM	2.4 g
dH2O	N/A	800 mL
**Adjust the pH to 7.4 with HCl and then add dH2O to 1 L**		
**Total**	**N/A**	**1 L**

**Note on storage conditions**: can be stored at room temperature (20°C–25°C) for months.

**Table:** 1 M Potassium phosphate buffer

Reagent	Final concentration	Amount
Monopotassium phosphate	136.1 g/L	136.1 g
Potassium hydroxide	17.99 g/L	17.99 g
dH2O	N/A	up to 1 L
**Total**	**N/A**	**1 L**

**Note on storage conditions**: can be stored at room temperature (20°C–25°C) for months.

**Table:** S-buffer

Reagent	Final concentration	Amount
Sodium chloride	5.84 g/L	2.92 g
1 M Potassium phosphate buffer (pH 6.0)	50 mM	25 mL
dH2O	N/A	475 mL
**Total**	**N/A**	**500 mL**

**Note on storage conditions**: can be stored at room temperature (20°C–25°C) for months.

**Table:** M9 buffer

Reagent	Final concentration	Amount
Monopotassium phosphate	12 g/L	48 g
Disodium phosphate	24 g/L	96 g
NaCl	20 g/L	80 g
dH2O	N/A	up to 4 L
**Total**	**N/A**	**4 L**

**Note on storage conditions**: can be stored at room temperature (20°C–25°C) for months.

**Table:** 0.5% PBS-Tween (PBS-T)

Reagent	Final concentration	Amount
10× PBS	1×	5 mL
Tween-20 100%	0.5%	250 μL
dH2O	N/A	up to 50 mL
**Total**	**N/A**	**50 mL**

**Note on storage conditions**: can be stored at room temperature (20°C–25°C) for months.

***Note:*** 100% Tween-20 is very viscous, and it may be hard to pipette small amounts accurately. For this reason, it may be necessary to prepare an intermediate dilution to ensure accurate pipetting.

**Table:** Monomer solution

Reagent	Final concentration	Amount
dH2O	N/A	258 μL
40% Acrylamide/Bis Solution 19:1	4%	30 μL
1 M Tris-HCl (pH 7.5)	40 mM	12 μL
**Total**	**N/A**	**300 μL**

**Note on storage conditions**: should be freshly prepared right before use and kept on ice.

**CRITICAL:** Acrylamide is neurotoxic. Gloves should be worn when handling acrylamide and it should be disposed of following appropriate hazardous waste-disposal procedures or polymerized before disposal in a regular trash.

**Table:** Bind-Silane pre-solution

Reagent	Final concentration	Amount
200 proof ethanol 100%	80%	800 mL
dH2O	18%	180 mL
Glacial acetic acid 100%	2%	20 mL
**Total**	**N/A**	**1 L**

**Note on storage conditions**: can be stored at room temperature (20°C–25°C) for months if the cap is tightly sealed with parafilm to avoid ethanol evaporation.

**Table:** Activated monomer solution

Reagent	Final concentration	Amount
dH2O	N/A	172 μL
40% Acrylamide/Bis Solution 19:1	4%	20 μL
1 M Tris-HCl (pH 7.5)	40 mM	8 μL
10% APS	0.05%	1 μL
10% TEMED	0.05%	1 μL
**Total**	**N/A**	**202 μL**

**Note on storage conditions**: should be freshly prepared right before use.

**CRITICAL:** Acrylamide is neurotoxic. Gloves should be worn when handling acrylamide and it should be disposed of following appropriate hazardous waste-disposal procedures or polymerized before disposal in a regular trash.

**Table:** 5% β-Mercaptoethanol solution

Reagent	Final concentration	Amount
dH2O	N/A	246 μL
1 M Tris-HCl (pH 7.5)	120 mM	36 μL
β-Mercaptoethanol 100%	5%	15 μL
Triton X-100 100%	1%	3 μL
**Total**	**N/A**	**300 μL**

**Note on storage conditions**: should be freshly prepared right before use.

***Note:*** 100% Triton X-100 is very viscous, and it may be hard to pipette small amounts accurately. For this reason, we suggest using an intermediate dilution to facilitate accurate pipetting.

**Table:** Collagenase buffer

Reagent	Final concentration	Amount
0.1 M CaCl2	50 mM	5 mL
1 M Tris-HCl (pH 7.5)	50 mM	500 μL
dH2O	N/A	4.5 mL
**Total**	**N/A**	**10 mL**

**Note on storage conditions**: can be stored at 4°C for months.

**Table:** Collagenase mix

Reagent	Final concentration	Amount
Collagenase	10 mg/mL	50 mg
Collagenase buffer	N/A	5 mL
**Total**	**N/A**	**5 mL**

**Note on storage conditions**: can be stored at −20°C for months.

**Table:** Blocking buffer

Reagent	Final concentration	Amount
5× PBS	1×	2 mL
BSA	50 mg/mL	0.5 g
Triton X-100 100%	0.5%	50 μL
NaN3 2%	0.05%	250 μL
0.5 M EDTA	1 mM	20 μL
dH2O	N/A	up to 10 mL
**Total**	**N/A**	**10 mL**

**Note on storage conditions**: can be stored at 4°C for a couple weeks.

**Table:** Primary and secondary antibody solution

Reagent	Final concentration	Amount
PBS 5×	1×	2 mL
BSA	10 mg/mL	0.1 g
Triton X-100 100%	0.5%	50 μL
NaN3 2%	0.05%	250 μL
EDTA 0.5 M	1 mM	20 μL
dH2O	N/A	Up to 10 mL
**Total**	**N/A**	**10 mL**

**Note on storage conditions**: carrier solution can be stored at 4°C for a couple weeks but the antibodies should only be added right before use. After adding the secondary antibody, the solution should be protected from light.

## Step-by-step method details

### Worm preparation and fixation

**Timing: 1.5–2 h**

The goal of this section is to harvest the worms and fix them. In this protocol we use 60% isopropanol to fix worms. However, due to the effect of fixatives on antigenicity,^2^ the optimal fixation condition may differ from one antibody to another.

1. Harvest ∼200–500 worms per immunostaining using S-buffer (or M9 buffer) in 1.5 mL tubes.
2. Centrifuge for 90 s at 400 × *g* and then aspirate the supernatant.
3. Resuspend in 1 mL of S-buffer (or M9 buffer).
4. Repeat steps 2 and 3 for a total of 2 washes. These steps are important to remove residual bacteria.
5. After the second wash, aspirate the supernatant and add 500 μL of 60% isopropanol.
6. Fix for a minimum of 10 min.

**Pause point:** Worms can be fixed and kept in 60% isopropanol at −20°C for at least a month.

7. Centrifuge for 90 s at 400 × *g*, and then aspirate the supernatant.
8. Resuspend in 500 μL 0.5% PBS-T.
9. Repeat steps 7 and 8 for a total of three washes.
10. Aspirate the 0.5% PBS-T after the third wash, and then resuspend the worms in 1 mL 1× PBS for 10 min.
11. Centrifuge for 90 s at 400 × *g* and aspirate the supernatant.
12. Add 300 μL of freshly prepared monomer solution and incubate the samples on a rocker or rotator at 4°C for at least an hour.

***Note:*** Samples can be left in the monomer solution overnight (12–16 h).

***Note:*** After fixation, worms become more prone to breaking. Therefore, worms should be allowed to settle either by gravity or through gentle centrifugation (<400 × *g*).

***Note:*** To save time, slide preparation may be performed during the ≥1 h incubation period (step 12).

**CRITICAL:** The monomer solution contains acrylamide, which is toxic, and should therefore be disposed of following appropriate hazardous waste-disposal procedures.

### Slide preparation

**Timing: <1 h**

The goal of this section is to prepare the slides used for the worm embedding. The polyacrylamide gel containing the embedded worms will be created by sandwiching the gel-worm mixture between two slides: (a) the Repel-Silane PTFE/Teflon slide, which will help sandwich the gel-worm mixture while avoiding gel and worms sticking to the glass; and (b) the Bind-Silane slide, where the gel, and therefore the worms, will stick to.

**Figure 1:**
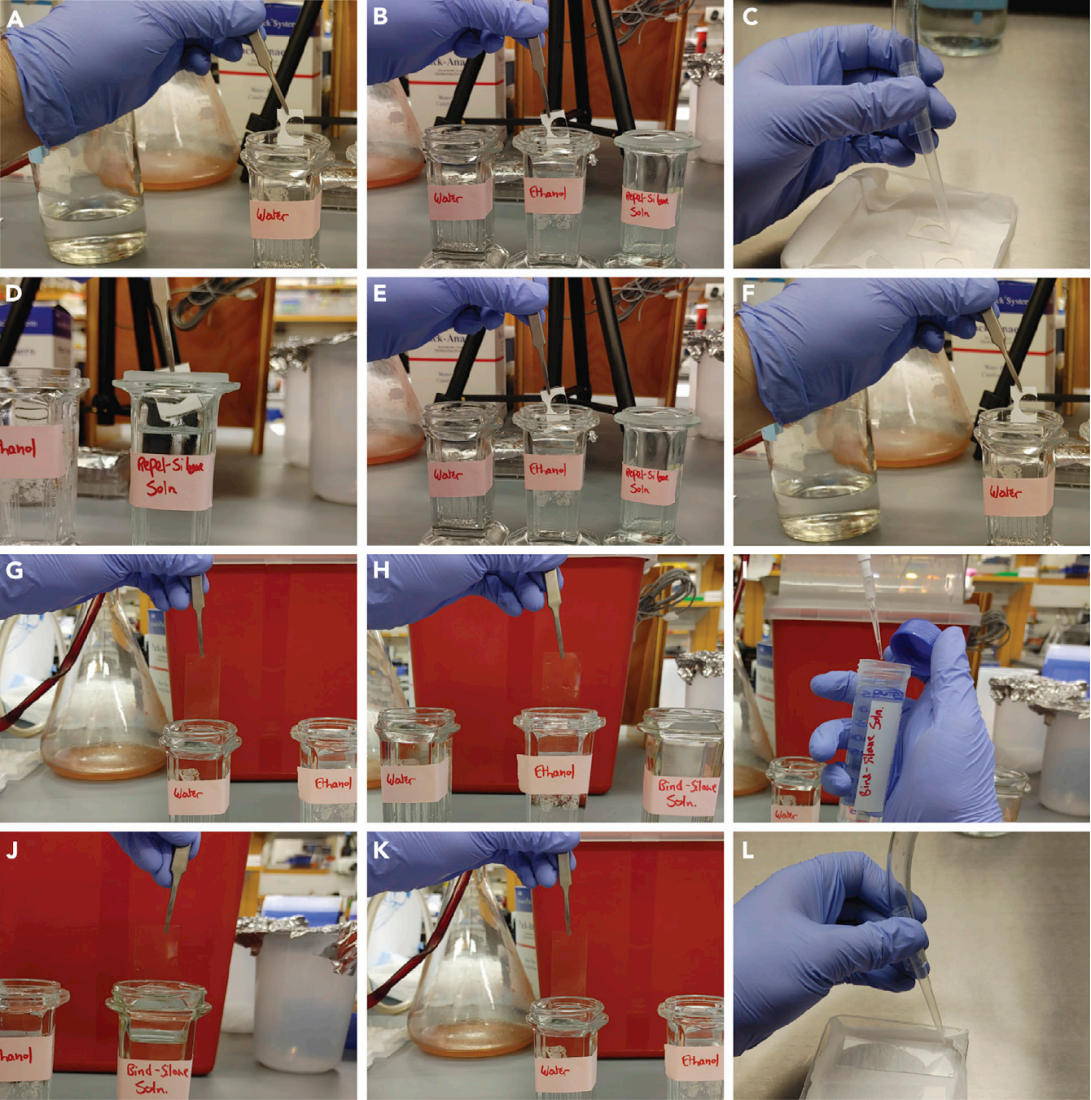
Preparation of Repel-Silane PTFE/TEFLON and Bind-Silane slides (A–L) Steps 13(a–g) and 14(a–g).
  b. Wash the PTFE/Teflon slides with freshly made 70% ethanol (Figure 1B).
  c. Use compressed air to dry the slides by blowing the air directly on the slides for about a minute (Figure 1C).
    ***Note:*** In case compressed air is not available the slides can be allowed to air dry however this may take a bit of time. We do not use Kim wipes to dry the slides to avoid lint.
    ***Note:*** It is critical for the slides to be clean, lint free, and dry.
  d. Incubate the PTFE/Teflon slides in Sigmacote or Repel-Silane for 15 s at room temperature (20°C–25°C) (Figure 1D).
  e. Air dry the slides at room temperature (20°C–25°C) for 5 min.
  f. Wash the slides with 70% ethanol followed by distilled water (Figures 1E and 1F).
  g. Air dry the slides at room temperature (20°C–25°C) and keep them in a closed box until needed.
    ***Note:*** Optional - for a more durable coating the slides can be oven dried for 1 h at 65°C.
    ***Note:*** Silanized slides can be stored at room temperature (20°C–25°C) for up to 1 month.

13. Repel-Silane PTFE/Teflon slide preparation:
  a. Wash the PTFE/Teflon slides with distilled water (Figure 1A).

14. Bind-Silane slide preparation:
  a. Wash standard microscope slides with distilled water (Figure 1G).
  b. Wash the slides with freshly made 70% ethanol (Figure 1H).
  c. Add 50 μL of Bind-Silane to 50 mL of previously prepared Bind-Silane pre-solution (Figure 1I).
  d. Incubate the slides in the Bind-Silane working solution for 30 s at room temperature (20°C–25°C) (Figure 1J).
  e. Air dry the slides at room temperature (20°C–25°C) for 5 min.
  f. Wash the slides with distilled water (Figure 1K).
  g. Use compressed air to dry the slides or allow the slides to air dry (Figure 1L).

***Note:*** Silanized slides can be stored at room temperature (20°C–25°C) for up to 1 month.

### Gel embedding

**Timing: 1–2 h**

In this section we describe how to embed the worms in the polyacrylamide gel. Additionally, we describe how to place the silicon gasket around the worms to ensure that the solutions used in the next steps do not leak out of the gel that contains the worms.

**Figure 2:**
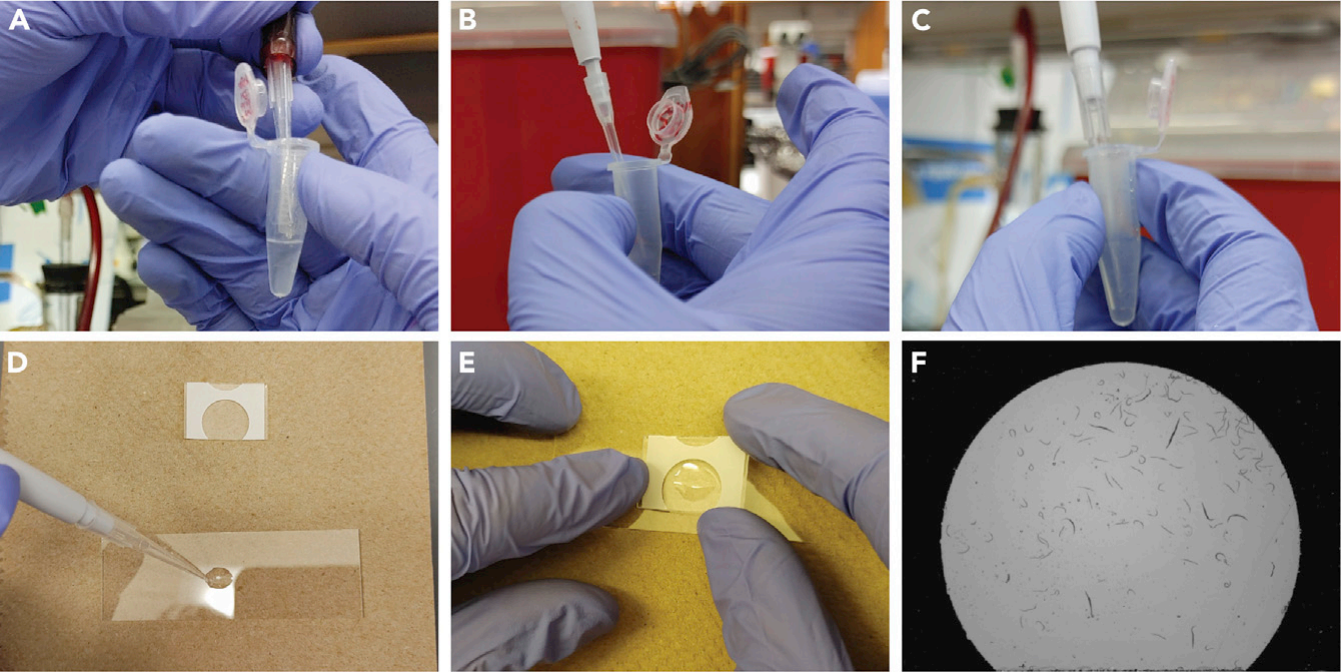
Preparation of gel-embedded worms (A–E) Steps 15–19. (F) A microscope image of the sandwiched worms.

15. Centrifuge the worms that were rocking at 4°C for 90 s at 400 × *g* and aspirate the monomer solution (Figure 2A).

**CRITICAL:** The monomer solution contains acrylamide, which is toxic and should therefore be disposed following appropriate hazardous waste-disposal procedures.

16. In a separate tube, activate the monomer solution by adding the appropriate amount of 10% APS and 10% TEMED (refer to Materials section) (Figure 2B).
17. Add 50 μL of activated monomer solution to the worms and resuspend gently by pipetting up and down (Figure 2C).

***Note:*** The activated monomer solution will remain liquid for at least an hour before solidifying. Nonetheless, we suggest that the APS and TEMED be added right before use.

18. Cast 25 μL of activated monomer solution on the Bind-Silane slide (Figure 2D).

***Note:*** Make sure not to transfer too many worms to avoid imaging overlapping specimens.

**Recommended numbers**: ∼50–100 adult worms or ∼100–200 L2 worms.

**Figure 3:**
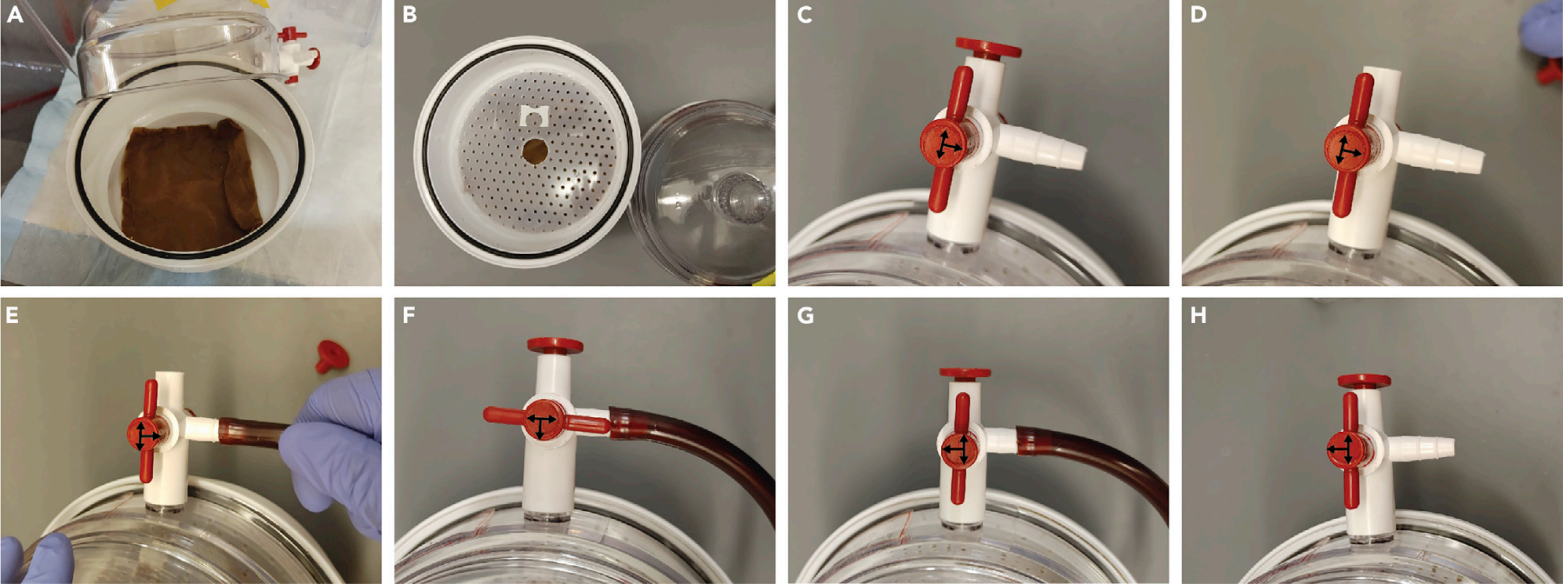
Setting-up vacuum chamber for gel polymerization (A) Place wet paper towels at the bottom of the vacuum chamber. (B) Place the platform in the chamber and your slide on top of the platform. (C) Place the dome on top to close the chamber. Note the direction of the valve with the arrows pointing towards the plug, the chamber, and the side opening. (D) Remove the plug. (E) Place the vacuum hose in the side opening and turn the vacuum on. (F) Turn the valve so that the arrows are pointing towards the opening where the hose was placed, the closed side, and the chamber. Put the plug back in and leave it in this position for around a minute. (G) Turn the valve so the arrows are now pointing to the plug, the chamber, and the closed side. (H) Turn off the vacuum and remove it from the side. Place the vacuum chamber in a 37°C incubator for 1 h. Black arrows were added for visual clarity. The arrows in the vacuum desiccator (Zoro #: G4673182) are red.

***Note:*** The polymerization is done in a vacuum chamber because oxygen inhibits free radical polymerization. Incubation can also be done in an argon or nitrogen atmosphere.

19. Sandwich the sample by placing the Repel-Silane (PTFE) slide on top of the Bind-Silane slide (Figure 2E).
20. Incubate the slides at 37°C for 1 h in a humidified vacuum chamber (Figure 3) to polymerize the gel.

21. Remove the sandwich from the chamber and place it on a clean workspace.
22. Carefully remove the Repel-Silane (PTFE) slide by lifting it using a razor blade (Figure 4A).

**Figure 4:**
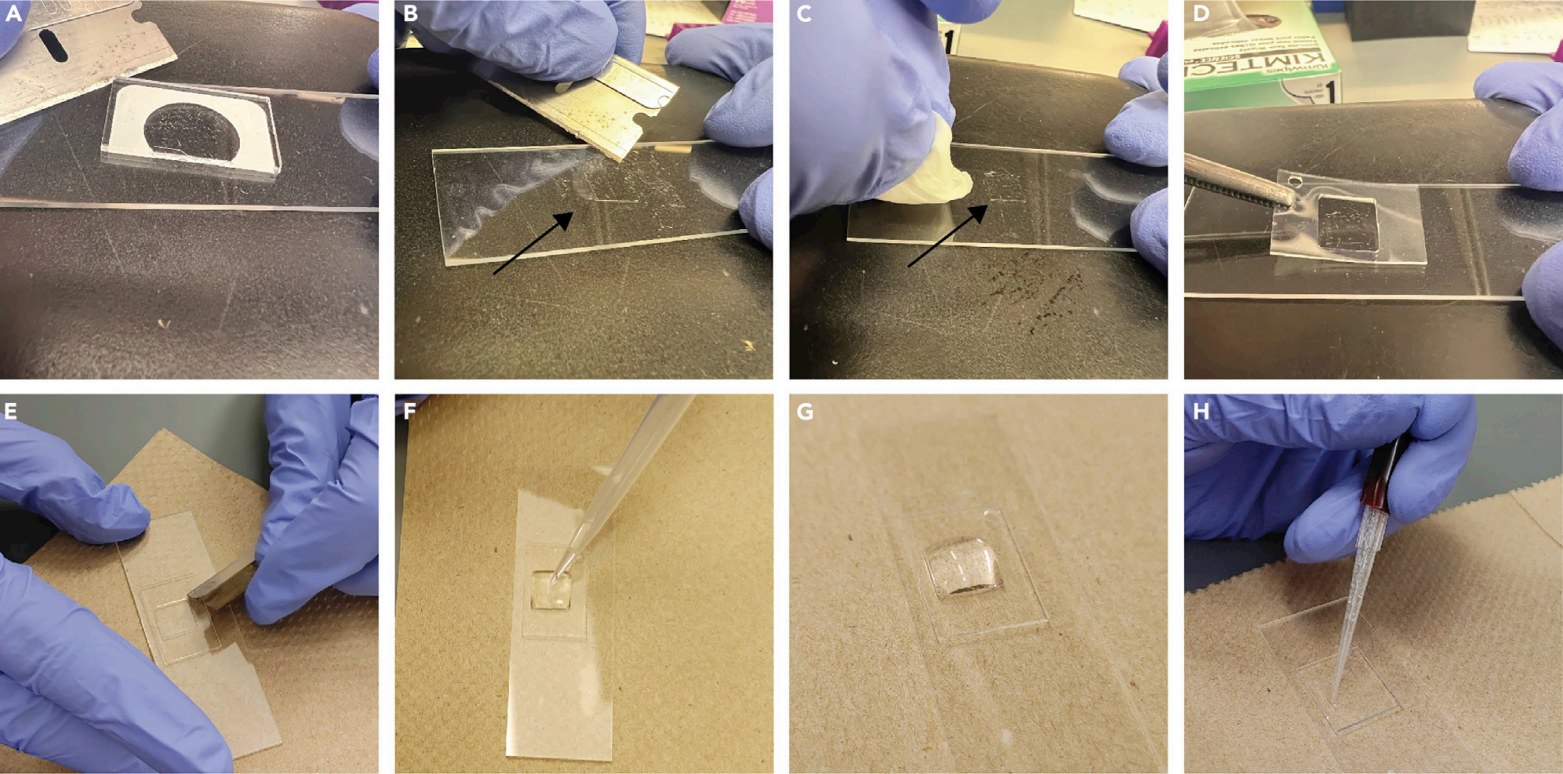
Preparing the slides for permeabilization & immunostaining (A–H) Steps 21–27. Arrows point to gel embedded worms.
23. Using the razor blade, cut the gel with the embedded worms (hereinafter referred to as the “sample gel”) into a rectangular shape estimated to fit tightly within the CultureWell silicon gasket (Figure 4B).

24. Before placing the sample gel in the CultureWell silicon gasket, smooth all edges using a sharp razor blade as described below in substeps a-b and as depicted in Figures 4B and 4C.
  ***Note:*** This cleaning step is critical to avoid leakage of liquid throughout the rest of the protocol.
  ***Note:*** Make sure not to create cracks in the slide by being forceful or by cleaning with the razor blade at the wrong angle.
  a. Use a razor blade at a 45° angle to remove all the excess gel around the area of interest to create a piece of sample gel that tightly fits into the silicon gasket and contains most of the embedded worms (Figure 4B).
  b. Once the excess gel is scraped off, use a Kimwipe with ethanol (200 proof) to thoroughly clean the area around the sample gel (Figure 4C).

25. Place the CultureWell silicon gasket around the sample while ensuring no air bubbles are created (Figure 4D).

***Note:*** If you do create bubbles while placing the gasket, either remove the gasket and try again or use the back of the razor to remove the bubble by pressing it across the surface of the gasket (Figure 4E).

***Note:*** The CultureWell silicon gasket can be cleaned with ethanol after the procedure and re-used. Store the silicon gasket by placing it between two microscope slides to avoid dust accumulation on the gasket which would affect its ability to stick to the slide. However, we have noticed that after several uses, the gasket tends to lose some of its stickiness.

26. Pipette 250 μL of 1× PBS into the well of the gasket and incubate for 5 min (Figures 4F and 4G).

### β-Mercaptoethanol and collagenase permeabilization

**Timing: 6 h**

In this section, we describe how to permeabilize the cuticle of the worm using β-mercaptoethanol and collagenase.

27. Aspirate the 1× PBS by placing the tip of the vacuum in the corner of the gasket (Figure 4H).

***Note:*** Do not touch the gel directly with the vacuum.

28. Add 250 μL of 5% BME solution and incubate for 2 h at 37°C in a humidified chamber.
29. Aspirate the BME solution and add 250 μL of 0.5% PBS-T.
30. Aspirate the PBS-T and add another 250 μL 0.5% PBS-T for a total of two washes.
31. Aspirate and add 250 μL of collagenase buffer and incubate for 5 min at room temperature (20°C–25°C).
32. Aspirate and add 250 μL of collagenase mix and incubate for 4 h at room temperature (20°C–25°C).

### Blocking and antibody staining

**Timing: 1 day**

In this section, we describe the incubation of the samples in the blocking solution which will prevent non-specific binding of the antibodies. Additionally, we describe the staining of the worms with a primary and secondary antibody.

33. Aspirate the collagenase and add 250 μL of blocking buffer for 5 min at room temperature (20°C–25°C).
34. Aspirate and repeat for a total of two 5 min incubations in the blocking buffer.

***Note:*** The length of the blocking step and the concentration of the blocking buffer may need to be optimized for the antibody and protein of interest. If the background signal observed at the imaging step is high, then the length of incubation in the blocking buffer and the percentage of BSA in the blocking buffer can be increased.

35. Aspirate the blocking buffer and incubate the samples in 250 μL of the primary antibody solution overnight (12–16 h) at 4°C.

**Figure 5:**
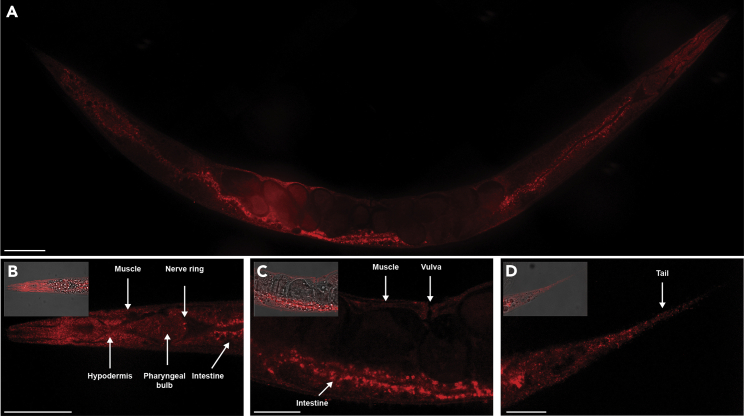
Immunostaining against the autophagy protein LGG-1 in adult *C. elegans* (A) Representative image of LGG-1 immunostaining across a whole-adult worm. (B) Anterior region of the adult worm. As previously reported,^3^ the most prominent signal in the head corresponds to the nerve ring. Additionally, immunostaining reveals strong LGG-1 signal in the pharynx, as well as anterior intestinal cells and muscle and hypodermal cells of the head. Inset depicts the corresponding DIC overlaid with the red channel. (C) Mid-body region of the worm. As previously described,^4^ intestinal cells show the most prominent autophagic signal in the *C. elegans* midbody. Immunostaining also confirms LGG-1 signal in the muscle cells of the midbody and the vulva, where LGG-1 expression had previously been reported.^5^ Inset depicts the corresponding DIC overlaid with the red channel. (D) Posterior area of the worm. As previously described,^6^ autophagic puncta are seen in the tail. Inset depicts the corresponding DIC overlaid with the red channel. (A–D) Wild type N2 worms were harvested as day-1 adults and processed as described above. The samples were incubated overnight (12–16 h) in rabbit anti-LGG-1 primary antibody (1:250) followed by a 2 h incubation in goat anti-rabbit antibody (1:500) (Invitrogen, Cat. #A11012). The images were taken with a Leica confocal microscope with an exposure time of 500 ms. Scale bar = 50 μm.

***Note:*** For the custom-made anti-LGG-1 rabbit antibody depicted in Figures 5 and 7 we used a 1/250 dilution, which is equivalent to 4.2 μg of antibody/mL. The concentration of primary antibody may need to be optimized based on the signal-to-noise ratio observed at the imaging step. If the signal at the imaging step is faint, then the primary antibody concentration may be increased.

36. The next day, aspirate the primary antibody solution and wash with 250 μL of the ‘primary antibody solution’ without the actual antibody.
37. Incubate for 5 min at room temperature (20°C–25°C).
38. Aspirate and repeat for a total of three washes in the ‘primary antibody solution’ (without the antibody).
39. Add 250 μL of the secondary antibody solution and incubate for 2 h at room temperature (20°C–25°C) in the dark.

***Note:*** After adding the secondary antibody, samples should be kept away from direct light as much as possible and all subsequent incubations should be performed in the dark.

**Figure 6:**
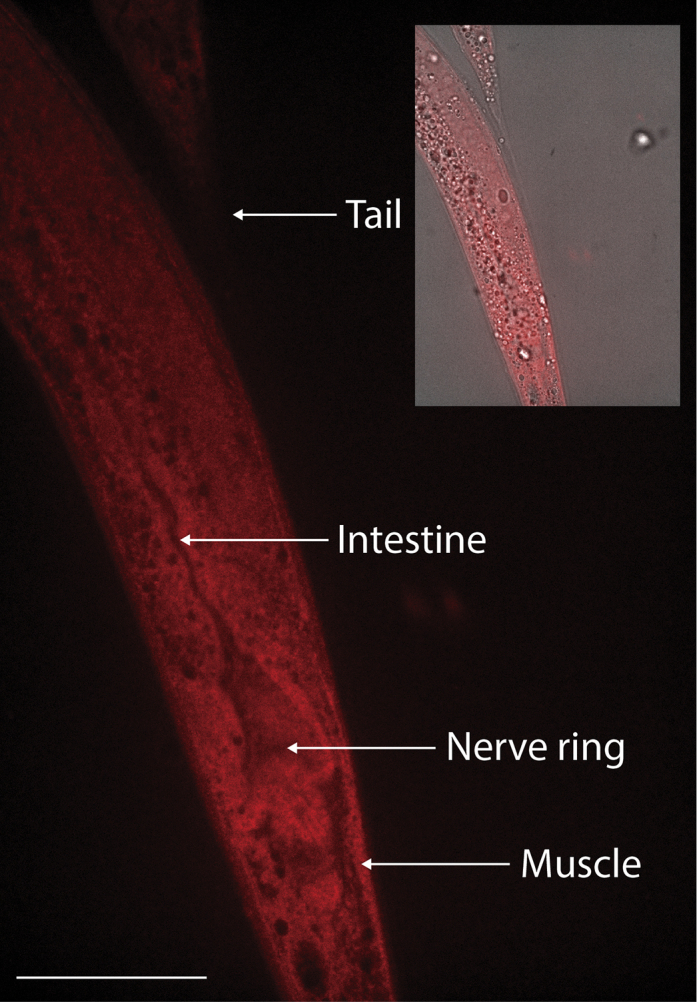
Worms stained with only the secondary antibody show no punctate structures Worms were harvested at day 1 of adulthood and processed as described above. The samples were incubated overnight (12–16 h) in the primary antibody solution but without the primary antibody, followed by a 2 h incubation in goat anti-rabbit secondary antibody (1:500). The images were taken with a Leica confocal microscope with an exposure time of 1 s (twice as long as the exposure time used for Figure 5). Unlike Figure 5, discrete punctate structures cannot be observed with the secondary antibody alone, demonstrating the specificity of the anti-LGG-1 immunostaining. Inset depicts the corresponding DIC overlaid with the red channel. Scale bar = 50 μm.

**Figure 7:**
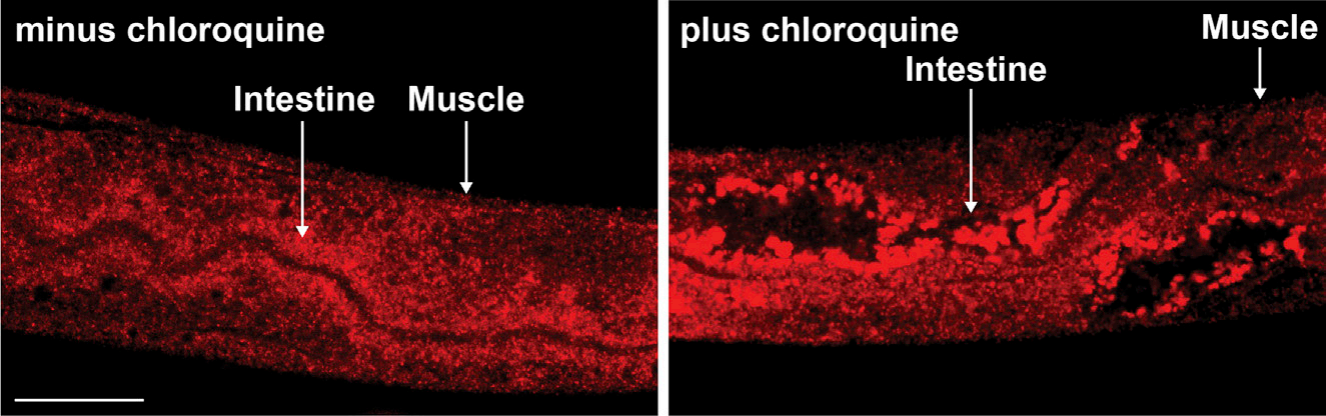
Chloroquine-treated *C. elegans* show coalescent LGG-1 signal The gel-embedded immunostaining approach enables visualization of the expected LGG-1 response to chloroquine: coalescence of signal. This result was expected from bulk biochemical studies in *C. elegans*^7^^,^^8^ and ample literature in other systems^9^ but the response of the endogenous LGG-1 has not been shown *in situ* for adult *C. elegans*. Wild-type 1-day adult worms were treated for 6 h in chloroquine 20 mM before being harvested in parallel with worms that were left untreated. Both chloroquine-treated (right panel) and untreated (left panel) worms were processed as described above and incubated overnight (12–16 h) in rabbit anti-LGG-1 primary antibody (1:250) followed by a 2 h incubation in goat anti-rabbit antibody (1:500) (Invitrogen, Cat. #A11012). The images were taken with a Leica confocal microscope and the exposure time for treated and untreated worms was 1 s. Scale bar = 50 μm.

***Note:*** For the LGG-1 staining depicted in Figures 5, 6, and 7 we used as secondary a 1/500 dilution of the Alexa Fluor 594 goat anti-rabbit antibody (Invitrogen, Cat. #A11012). The concentration of the secondary antibody may need to be optimized based on the signal-to-noise ratio observed at the imaging step.

40. Aspirate and wash with 250 μL of the ‘secondary antibody solution’ (without the actual antibody).

***Note:*** Optional – for nuclear staining, add 100ng/mL DAPI or Hoechst to the ‘secondary antibody solution’.

***Note:*** Optional – additional washing may be necessary if the background signal is too high (refer to problem 5 in the troubleshooting section).

41. Incubate for 5 min at room temperature (20°C–25°C).

***Note:*** Optional – if staining nuclei incubate for 30 min at room temperature (20°C–25°C).

42. Aspirate the solution and then remove the CultureWell silicon gasket.
43. Clean the slide with a Kimwipe with ethanol (200 proof).
44. Add a drop of mounting media (e.g., Vectashield SKU:H-1000-10).
45. Place a coverslip on top and seal it with nail polish.
46. Proceed to fluorescent imaging.

## Expected outcomes

We used this procedure to stain autophagic puncta by immunostaining against the ubiquitously expressed autophagy protein LGG-1 (Figure 5). The images demonstrate that the procedure presented here enables antibody staining without disrupting the anatomical structure of *C. elegans* (Figures 5A–5D). Additionally, the LGG-1 signal can be observed across tissues. This is illustrated by zoomed-in images showing LGG-1 expression in the nerve ring (Figure 5B), the body-wall muscle and intestine (Figures 5B and 5C), the vulva (Figure 5C), and the tail (Figure 5D). Importantly, we did not observe puncta-like signal when immunostaining worms with the secondary antibody only (Figure 6). To test the extent to which the method can detect a subcellular physiological response, we also immunostained worms treated with the autophagy inhibitor chloroquine, which causes accumulation and enlargement of the autophagic puncta.^9^ As expected, we observed coalescence of autophagic puncta post-chloroquine treatment (Figure 7).

## Limitations

The fixation, permeabilization and antibody staining methods used here are used in classical *C. elegans* immunohistochemistry. Therefore, they have been used for staining of many proteins.^2^ We don’t anticipate the gel embedding to interfere with the staining of proteins that are otherwise compatible with these reagents.

On the other hand, the permeabilization of worms using β-Mercaptoethanol and collagenase has previously been shown to disrupt certain antigens.^2^ Proteins that are incompatible with these treatments (or any chemical and enzymatic treatment used for cuticle permeabilization) would have to be visualized using the freeze-crack method.^10^

## Troubleshooting

In step 32 of this protocol, we use Collagenase type I for the permeabilization of the *C. elegans* cuticle, however previous studies have successfully used other collagenases for the permeabilization including Collagenase type VII^2^ and Collagenase type IV.^11^ Although we haven’t tried other collagenases, we suspect that it is possible to replace Collagenase type I with other forms.

The goal of the Silicon gasket is to ensure that the liquid remains on the samples and does not dissipate across the slide. We have tried using hydrophobic barrier pens for that purpose with little success.

### Problem 1

The gel did not polymerize in step 20.

### Potential solution

This could be caused by the absence or use of a low concentration of APS and/or TEMED. This could also be caused by a leakage in the vacuum desiccator in step 20. Make sure that the dome of the desiccator is placed properly, and that the stopcock is fully inserted.

### Problem 2

Leakage of liquid through the silicon gasket.

### Potential solution

Make sure the area around the gel is thoroughly cleaned before placing the silicon gasket (step 20). Leakage could also be caused by bubbles formed during the placement of the silicon gasket (step 25), so make sure to place the silicon gasket carefully and remove any bubbles by rolling air out of the gasket (e.g., with the back of the razor blade).

### Problem 3

Worms cannot be imaged fully since they are overlapping each other.

### Potential solution

Reduce the number of worms placed on the slide (step 18).

### Problem 4

No signal after antibody staining.

### Potential solution

This could have several causes:

• One possible cause would be not using hydrated chambers in steps 20 and 28.
• The concentration of primary and secondary antibodies needs to be optimized.
• For proteins that are sensitive to the chemical (β-mercaptoethanol) and enzymatic (collagenase) treatments employed here, the length of the treatments and the concentration of the reagents may need to be adjusted. Additionally, β-mercaptoethanol and collagenase could, theoretically, be substituted by other chemicals able to permeabilize the cuticle of the worms.

### Problem 5

High background signal.

### Potential solution

Increase the length of incubation in the blocking buffer and/or the percentage of BSA used in the blocking buffer (a typical range would be 0.3%–5%). Another potential solution would be to decrease the concentration of the primary or secondary antibodies. Users may also want to increase the number of washes after incubation in the secondary antibody or try different secondary antibodies since some may be associated with a high background signal. Finally, every time a new primary antibody is used, it is important to run a secondary antibody-only control to determine background signal.

## Resource availability

### Lead contact

Further information and requests for resources and reagents should be directed to and will be fulfilled by the Lead Contact, Eyleen J. O’Rourke (ejorourke@virginia.edu).

### Materials availability

This study did not generate new unique reagents.

## Data Availability

This study did not generate or analyze datasets and codes.
